# Nomogram for the prediction of crescent formation in IgA nephropathy patients: a retrospective study

**DOI:** 10.1186/s12882-023-03310-2

**Published:** 2023-09-04

**Authors:** Zaoqiang Lin, Liuchang Feng, Huan Zeng, Xuefei Lin, Qizhan Lin, Fuhua Lu, Lixin Wang, Jianling Mai, Pingjun Fang, Xusheng Liu, Qinxiang Tan, Chuan Zou

**Affiliations:** 1 Department of Nephrology, Shenzhen Hospital, Beijing University of Chinese Medicine, Shenzhen, China; 2 Department of Nephrology, Jiujiang Hospital of Traditional Chinese Medicine, Jiujiang, China; 3 Department of Nephrology, Guangdong Provincial Hospital of Chinese Medicine, Guangzhou, China; 4 Department of Hemodialysis, Guangzhou Charity Hospital, Guangzhou, China; 5 Department of Hemodialysis, Guangdong Provincial Hospital of Chinese Medicine, Guangzhou, China

**Keywords:** IgA nephropathy, Crescent, Prediction, Nomogram

## Abstract

**Background:**

The 2017 Oxford classification of immunoglobulin A nephropathy (IgAN) recently reported that crescents could predict a worse renal outcome. Early prediction of crescent formation can help physicians determine the appropriate intervention, and thus, improve the outcomes. Therefore, we aimed to establish a nomogram model for the prediction of crescent formation in IgA nephropathy patients.

**Methods:**

We retrospectively analyzed 200 cases of biopsy-proven IgAN patients. Least absolute shrinkage and selection operator(LASSO) regression and multivariate logistic regression was applied to screen for influencing factors of crescent formation in IgAN patients. The performance of the proposed nomogram was evaluated based on Harrell’s concordance index (C-index), calibration plot, and decision curve analysis.

**Results:**

Multivariate logistic analysis showed that urinary protein ≥ 1 g (OR = 3.129, 95%CI = 1.454–6.732), urinary red blood cell (URBC) counts ≥ 30/ul (OR = 3.190, 95%CI = 1.590–6.402), mALBU ≥ 1500 mg/L(OR = 2.330, 95%CI = 1.008–5.386), eGFR < 60ml/min/1.73m^2^(OR = 2.295, 95%CI = 1.016–5.187), Serum IgA/C3 ratio ≥ 2.59 (OR = 2.505, 95%CI = 1.241–5.057), were independent risk factors for crescent formation. Incorporating these factors, our model achieved well-fitted calibration curves and a good C-index of 0.776 (95%CI [0.711–0.840]) in predicting crescent formation.

**Conclusions:**

Our nomogram showed good calibration and was effective in predicting crescent formation risk in IgAN patients.

## Introduction

IgA nephropathy (IgAN) is the most common form of primary glomerulonephritis. It has been reported that 25–40% of patients will develop end-stage renal disease (ESRD) within 10–20 years [1]. Due to the clinical and pathological diversity of IgAN,this disease progression and prognosis varies interindividually [2, 3]. The pathological features of IgAN is highly variable, such as renal active lesions(i.e., crescent formation) and chronic glomerular pathology features(i.e., tubular-interstitial fibrosis).

Crescent formation is a common histopathological finding,occurring in approximately 20–60% of IgAN patients [4]. Although the original Oxford study and several subsequent validation studies did not find the predictive value of crescents [5, 6], an increasing number of studies have found that crescents to be an independent predictor of poor renal outcomes in patients with IgAN [7, 8]. Recently, a multicenter study conducted by Haas and colleagues evaluated the effect of crescents on renal function progression [9]. Based on 3,096 IgAN patients from four retrospective studies across the world, crescents were found to be strong predictors of unfavorable prognosis of IgAN. Consequently, cellular and/or fibrocellular crescents (C) were include in the updated Oxford classification of IgAN:C0(no crescents), C1(crescents in < 25% of glomeruli), and C2(crescents in ≥ 25% of glomeruli) [10].

Considering that the glomerular crescent is highly associated with poor renal prognosis of IgA nephropathy, early diagnosis and intervention of crescent is necessary. Although renal biopsy is the gold standard for the diagnosis of IgAN, it is an invasive operation that is prone to complications such as bleeding and infection, which is not acceptable for some patients [11, 12]. In addition, repeat biopsies in IgAN are still remain rare and it is sometimes difficult to judge the acute and chronic renal lesions of an individual patient, which makes it difficult to use the time-to-event data of the population to guide treatment. Hence, it is clinically necessary to establish a safe and non-invasive diagnostic method in biopsy-proven IgAN patients to predict the risk of developing crescents for as an alternative to re-biopsy. However, the individualized prediction of IgA nephropathy with crescent has been rarely reported and should be urgently solved. Therefore, the purpose of this study was to examine influencing factors of crescent formation and construct a nomogram for predicting the incidence of the crescent in IgAN patients.

## Methods

### Study design and population

This retrospective cohort study included biopsy-proven IgAN patients hospitalized in Guangdong Provincial Hospital of Chinese Medicine between May 2005 and November 2017. The inclusion criteria were: (1) 18 years of age or older; (2) patients with biopsy-proven primary IgAN; (3) With less than eight glomeruli on light microscopy. The exclusion criteria were: (1) insufficient clinical and pathological data; (2) atypical IgA nephropathy, such as crescentic GN; (3) Individuals diagnosed secondary IgAN such as rheumatology disease, Henoch–Schonlein purpura.or liver cirrhosis.

Ethical approval was granted by the Ethics Committee of Guangdong Provincial Hospital of Chinese Medicine (B2016-155-01). Informed consent was exempted since the study only involved analysis of anonymized existing data and records.

### Data collection and definition

In this cohort, we recorded demographic variables and laboratory indicators during kidney biopsy, including gender, body mass index(BMI), 24 h urinary protein excretion, immunoglobulin A and complement 3 (C3). eGFR was calculated by the CKD-EPI equation [13]. MAP was equal to diastolic BP + 1/3(systolic BP - diastolic BP) [14]. Medications that block the renin-angiotensin system(RAAS) and immunosuppression( mainly steroid therapy, and other drugs) were recorded after kidney biopsy.

The 2017 updated Oxford Classification (MEST-C) was used in this study [10]: mesangial hypercellularity (M):M1 was defined as a mesangial hypercellularity score > 0.5. Endocapillary hypercellularity (E): E1 was defined as the presence of endocapillary hypercellularity. Segmental glomerulosclerosis (S):S1 was defined as the presence of segmental glomerulosclerosis. Tubular atrophy/interstitial fibrosis (T):T1 was defined as tubular atrophy/interstitial fibrosis within 26–50% of the cortical area, and T2 was defined as tubular atrophy/interstitial fibrosis in > 50% of the cortical area. Crescent(C):C1 was defined as crescents in < 1/4 of all glomeruli, and C2 was defined as crescents in ≥ 1/4 of all glomeruli.

### Statistical analysis

Missing data was imputed using multiple imputation with chained equations to generate 5 complete datasets, with results combined across imputed datasets. We have adopted the existing sample size criteria by Riley et al. [15] and pmsampsize R library [16, 17] to calculate the sample size. On the basis of a study by Lee M J et al. [18], it was expected that the prevalence of crescents in IgAN patients is 20%. We estimated a C-index of 0.8 for this prediction model and planned to collect 22 predictor variables. To ensure an accurate estimate of overall risk (model intercept), at least 246 subjects were required. However, since our study was a single-center retrospective study and the incidence of IgAN patients at the center was not high, the sample size was derived on the basis of the available data. Pearson’s test and Chi-square test were used to test the distribution of categorical variables; the Mann-Whitney U test and paired-sample t-test were used for continuous variables. LASSO regression was used to screen for optimal risk factors of crescent formation in IgA patients. Stepwise logistic regression model was used build a prediction model by introducing the features selected in the LASSO regression model and the β regression coefficient, odds ratio (OR), and P-value were calculated. Additionally, the forest plot was drawn to describe the P-value, OR and 95% CI of selected validation visually. A nomogram was constructed to predict the possibility of crescents formation based on statistically significant factors identified by the multivariate logistic regression model. The predictive performance of the nomogram was evaluated using Harrell’s concordance index (C-index), and calibration with 1000 bootstrap samples was performed to decrease the overfit bias. The predictive accuracy and diagnostic performance of the nomogram were quantified using the areas under the ROC curves. Decision curve analysis (DCA) was used to determine the clinical practicability of nomograms based on the net benefit according to different threshold probabilities in IgAN patients. Statistical analyses were conducted using SPSS 22.0 (IBM Corp, Armonk, NY, USA) and the R software (version 3.6.2; https://www.R-project.org). All reported p values were single-sided; statistical significance was considered at p < 0.05.

## Results

### Characteristics of cohort

All 200 IgA nephropathy patients were divided into the C0 group (118 cases) and C ≥ 1 group (82 cases) according to the presence or absence of a crescent. Among these participants, there were 106 females (53%) and 94 males (47%), with a median age of 32 (26–42) years and MAP of 94.67 (87.33-104.58) mmHg. The C ≥ 1 group was characterized by a significantly higher rate of urinary protein ≥ 1 g, URBC counts ≥ 30/ul, eGFR < 60ml/min/1.73m^2^ and serum IgA/C3 ratio ≥ 2.59. As for urinary protein components, patients in the C ≥ 1 group had higher levels of IgGU[80.40(35.78-207.75) vs. 33.05(15.78–83.8), p < 0.001], α1-MgU[12.8(6.29–28.63) vs. 9.09(5.56–17.33), p = 0.005], α2-MgU[2.55(2.34–5.32) vs. 2.41(2.39–2.55), p = 0.023], TrfU[48.9(20-131.5) vs. 17.5(8.47–48.48), p = 0.023], and higher proportion of mALBU ≥ 1500 mg/L(43.9% vs. 14.4%, p < 0.001). However, indicators as sex, BMI, TG, UA presented no differences between the two groups (P > 0.05). (Table 1).

**Table 1:** Clinical and histological characteristics

Variables	Total (n = 200)	C0 group (n = 118)	C ≥ 1 group (n = 82)	*P-value*
Female ,n(%)	106(53)	59(50)	47(57.3)	0.308
Age (years)	32(26–42)	32.5(25.7–42)	31(26–46)	0.563
MAP (mmHg)	94.67(87.33-104.58)	92.83(86.67-100.67)	97.67(88.33-107.42)	0.009
BMI(kg/m^2^)	22(19.82–24.61)	21.79(19.61–24.52)	22.48(20.25–24.80)	0.519
Urinary protein (g/24 h) (%)				< 0.001
< 1 g	108(54)	80(67.8)	28(34.1)	
≥ 1 g	92(46)	38(32.2)	54(65.9)	
URBC (counts /ul) (%)				0.003
< 30	78(39)	56(47.5)	22(26.8)	
≥ 30	122(61)	62(52.5)	60(73.2)	
eGFR (ml/min/1.73m^2^)(%)				0.017
< 60	158(79)	100(84.7)	58(70.7)	
≥ 60	42(21)	18(15.3)	24(29.3)	
UA(mmol/L)	399.00 ± 107.16	390.09 ± 101.11	411.84 ± 114.72	0.159
TC(mmol/L)	4.73(4.13–5.55)	4.64(4.02–5.35)	5.15(4.22–5.80)	0.029
TG(mmol/L)	1.38(0.91–1.95)	1.25(0.83–1.86)	1.57(0.98–2.09)	0.069
TP(g/L)	66.15(60.2–70.9)	67.4(62.35–71.68)	64.71(57.18–69.93)	0.065
ALB (g/L)	40.05(36.13–42.9)	40.3(37.13–43.13)	38.2(33.1-42.65)	0.039
BUN(mmol/L)	5.15(4.28–6.40)	4.94(4.35–5.95)	5.41(4.15–7.29)	0.072
LDL(mmol/L)	3.01(2.48–3.69)	2.82(2.42–3.55)	3.26(2.63–3.82)	0.016
HDL(mmol/L)	1.19(0.95–1.49)	1.21(0.95–1.45)	1.17(1.01–1.57)	0.915
Serum IgA/C3 (%)				0.024
< 2.59	72(36)	50(42.4)	22(26.8)	
≥ 2.59	128(64)	68(57.6)	60(73.2)	
IgGU(mg/L)	50.65(21.75-123.25)	33.05(15.78–83.80)	80.40(35.78-207.75)	< 0.001
β2-MgU (mg/L)	0.23(0.22–0.40)	0.23(0.22–0.32)	0.23(0.22–0.73)	0.152
mALBU (mg/L)(%)				< 0.001
< 1500	147(73.5)	101(85.6)	46(56.1)	
≥ 1500	53(26.5)	17(14.4)	36(43.9)	
α1-MgU (mg/L)	10.02(5.56–20.9)	9.09(5.56–17.33)	12.80(6.29–28.63)	0.005
α2-MgU(mg/L)	2.47(2.37–2.66)	2.41(2.39–2.55)	2.55(2.34–5.32)	0.023
TrfU(mg/L)	29.45(11.25–74.53)	17.50(8.47–48.48)	48.9(20-131.50)	< 0.001
Oxford classification				
M1%	181(90.5)	100(84.7)	81(98.8)	0.001
E1%	33(16.5)	7(5.9)	26(31.7)	< 0.001
S1%	118(59)	63(53.4)	55(67.1)	0.053
T1 + 2%	60(30)	23(19.5)	37(45.1)	< 0.001
Treatment				
With RAAS blockade	133(66.5)	80(67.8)	53(64.6)	0.641
With immunosuppression	23(11.5)	9(7.6)	14(17.1)	0.039

Values are expressed as mean ± SD, medians (interquartile ranges), or percentages.

MAP, mean arterial pressure; BMI, body mass index; URBC, urinary red blood cell; eGFR, estimated glomerular filtration rate; BUN, Blood Urea Nitrogen; LDL, Low-density Lipoprotein; HDL, High-density Lipoprotein; IgGU, urine immunoglobulin G; β2-MgU, urine β2-microglobulin; mALBU, urine micro-albumin; α1-MgU, urine α1-microglobulin; α2-MgU, urine α2-microglobulin; TrfU, Urinary transferrin.

As for MEST-C score, patients in the C ≥ 1 group had the higher the proportion of M1 (98.8% vs. 84.7%, p = 0.001), E1 (31.7% vs. 5.9%, p < 0.001), and T1 + 2 (45.1% vs. 19.5%, p < 0.001). Immunosuppression were more commonly used in the C ≥ 1 group than in the C0 group (17.1% vs. 7.6%, p = 0.039).

### Characteristics selection

**Fig. 1:**
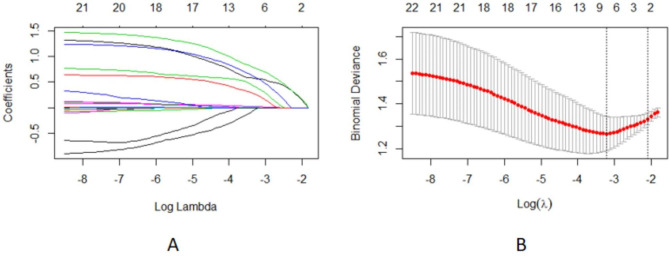
Variables selection using the LASSO binary logistic regression model. **(a)** Optimal parameter (lambda) selection in the LASSO model used 5-fold cross-validation via minimum criteria. The partial likelihood deviance (binomial deviance) curve was plotted versus log (lambda). Dotted vertical lines were drawn at the optimal values by using the minimum criteria and the 1 SE of the minimum criteria. **(b)** LASSO coefficient profiles of the 22 variables. A coefficient profile plot was produced against the log (lambda) sequence. 5 variables with nonzero coefficients were selected by optimal lambda

In terms of clinical features, we used LASSO regression to identify the main variables related to crescent formation in IgAN patients, and a total of 5 variables were screened out of 22 features. These variables included urinary protein ≥ 1 g, URBC counts ≥ 30/ul, mALBU ≥ 1500 mg/L, eGFR < 60ml/min/1.73m^2^ and serum IgA/C3 ratio ≥ 2.59 **(**Fig. 1a **and b)**.

**Fig. 2:**
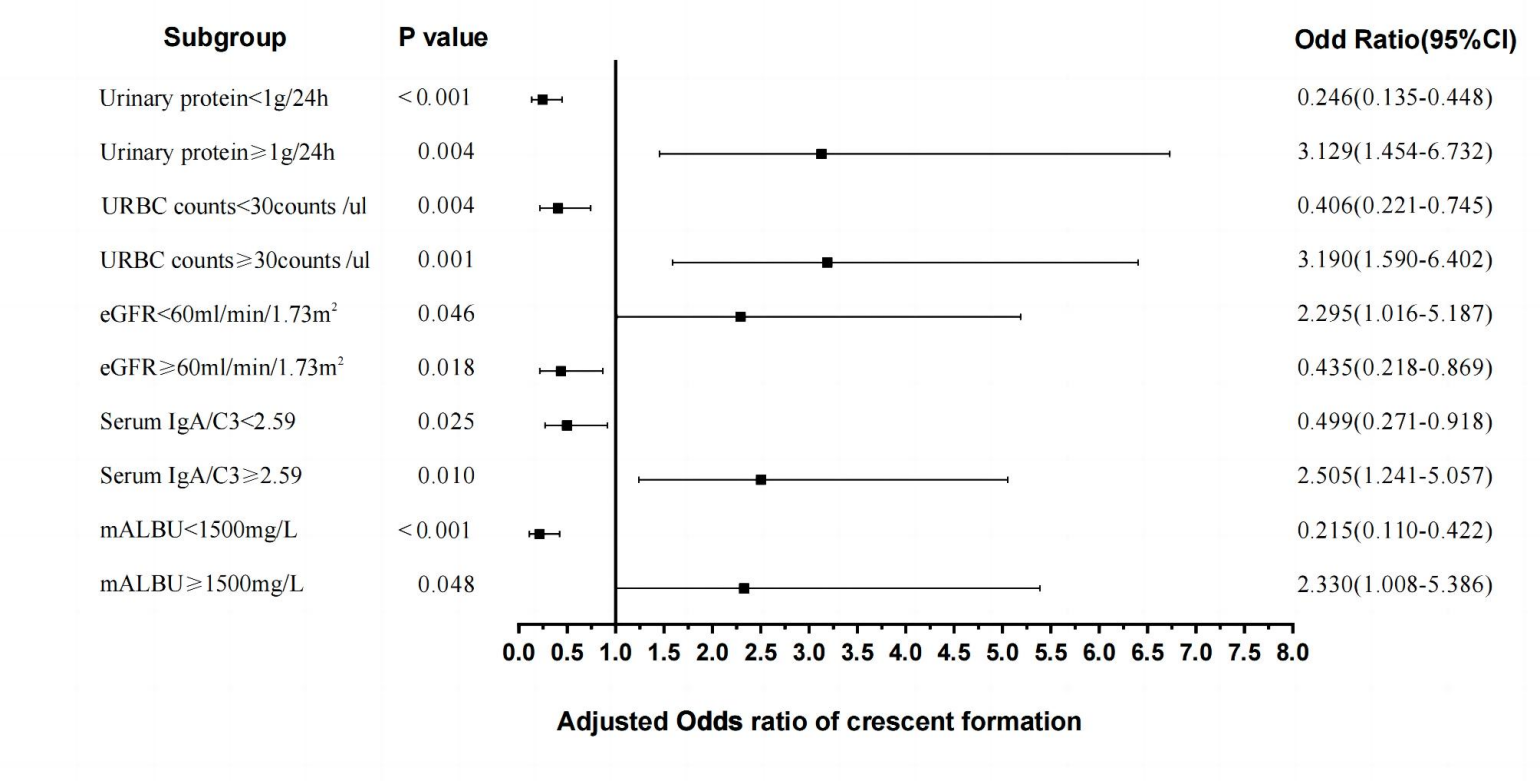
The forest plot of the OR of the selected variables. Forest plot for outcome in LASSO regression model and multivariate logistic regression analysis. URBC counts, Urinary red blood cell counts; eGFR, Estimated glomerular filtration rate; mALBU, urine micro-albumin

Next, candidate predictors variables selected by LASSO regression were included in stepwise logistic regression analysis. The stepwise analysis showed that urinary protein ≥ 1 g (OR = 3.129; 95%CI [1.454–6.732]; P = 0.004), serum IgA/C3 ratio ≥ 2.59 (OR = 2.505; 95%CI [1.241–5.057]; P = 0.010), URBC counts ≥ 30/ul (OR = 3.190; 95%CI [1.590–6.402]; P = 0.001), mALBU ≥ 1500 mg/L (OR = 2.330; 95%CI [1.008–5.386]; P = 0.048) and eGFR < 60ml/min/1.73m^2^ (OR = 2.295; 95%CI [1.016–5.187]; P = 0.046) were associated with incident crescent formation (Table 2). Based on LASSO regression and stepwise logistic regression to exclude overfitting and multicollinearity, five factors were included in the prediction model, and the forest plot summary is shown in Fig. 2.

**Table 2:** Multivariate logistic regression analysis

Variables	Univariable		Multivariable	
	OR (95% CI)	P value	OR (95% CI)	P-value
Female	1.343(0.762–2.368)	0.308		
Age (years)	1.013(0.988–1.039)	0.321		
MAP (mmHg)	1.028(1.005–1.052)	0.017		
BMI(kg/m^2^)	1.002(0.922–1.088)	0.965		
Urinary protein ≥ 1 g (g/24 h)	4.060(2.233–7.382)	< 0.001	3.129(1.454–6.732)	0.004
URBC counts ≥ 30/ul	2.463(1.342–4.523)	0.004	3.190(1.590–6.402)	0.001
eGFR<60ml/min/1.73m^2^	2.299(1.151–4.590)	0.018	2.295(1.016–5.187)	0.046
UA(mmol/L)	1.002(0.999–1.005)	0.159		
TC(mmol/L)	1.151(0.960–1.379)	0.128		
TG(mmol/L)	1.176(0.921–1.501)	0.194		
TP(g/L)	0.979(0.949–1.010)	0.176		
ALB (g/L)	0.962(0.922–1.002)	0.065		
BUN(mmol/L)	1.228(1.063–1.419)	0.005		
LDL(mmol/L)	1.143(0.920–1.420)	0.226		
HDL(mmol/L)	1.024(0.492–2.132)	0.950		
Serum IgA/C3 ≥ 2.59	2.005(1.090–3.690)	0.025	2.505(1.241–5.057)	0.010
IgGU(mg/L)	1.005(1.002–1.007)	0.002		
β2-MgU(mg/L)	1.229(1.027–1.471)	0.025		
mALBU ≥ 1500 mg/L	4.650(2.370–9.122)	< 0.001	2.330(1.008–5.386)	0.048
α1-MgU(mg/L)	1.019(1.003–1.035)	0.021		
α2-MgU(mg/L)	1.074(1.006–1.146)	0.032		
TrfU(mg/L)	1.002(0.999–1.005)	0.171		

MAP, mean arterial pressure; BMI, body mass index; URBC, urinary red blood cell; eGFR, estimated glomerular filtration rate; BUN, Blood Urea Nitrogen; LDL, Low-density Lipoprotein; HDL, High-density Lipoprotein; IgGU, urine immunoglobulin G; β2-MgU, urine β2-microglobulin; mALBU, urine micro-albumin; α1-MgU, urine α1-microglobulin; α2-MgU, urine α2-microglobulin; TrfU, Urinary transferrin.

### Development of an individualized prediction model

**Fig. 3:**
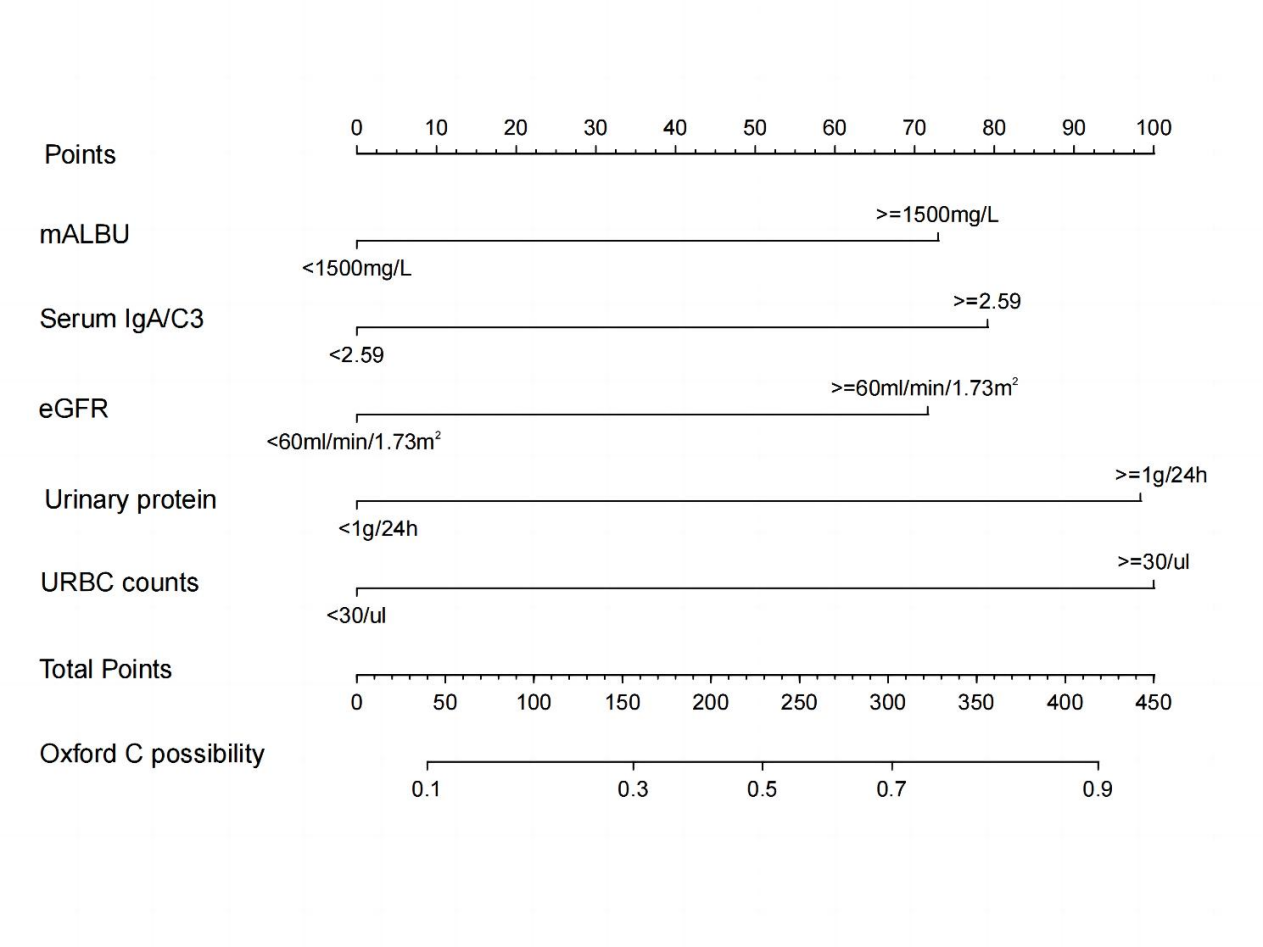
Nomogram for predicting of crescent formation in IgA nephropathy patients. The crescent formation nomogram was developed in the cohort, with five variables, urinary protein, URBC counts, mALBU, eGFR and serum IgA/C3. Abbreviations: URBC counts, Urinary red blood cell counts; eGFR, Estimated glomerular filtration rate; mALBU, urine micro-albumin

A model containing the above independent predictors was developed and presented as a nomogram (Fig. 3). The scores of different variables were obtained on the vertical line on the nomogram, after which the total risk score was calculated by adding all the scores of all variables. The probability of crescent formation could be directly read on the total point axis.

### Validation of prediction model

**Fig. 4:**
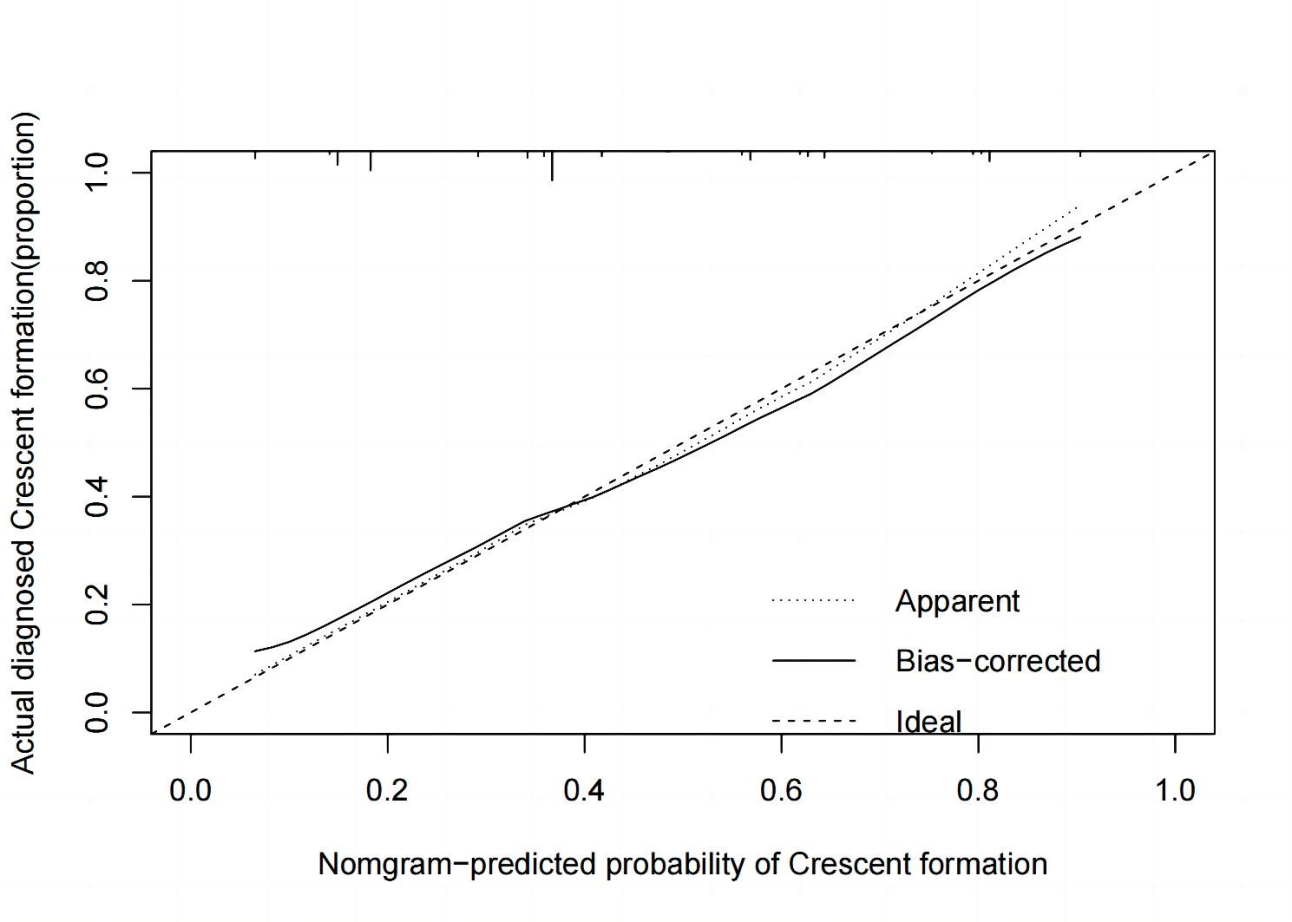
Calibration curve for the crescent formation nomogram prediction in the cohort. The x-axis represents the predicted crescent formation risk. The y-axis represents the actual diagnosed crescent formation. The diagonal dotted line represents a perfect prediction by an ideal model. The solid line represents the performance of the nomogram, of which a closer fit to the diagonal dotted line represents a better prediction

**Fig. 5:**
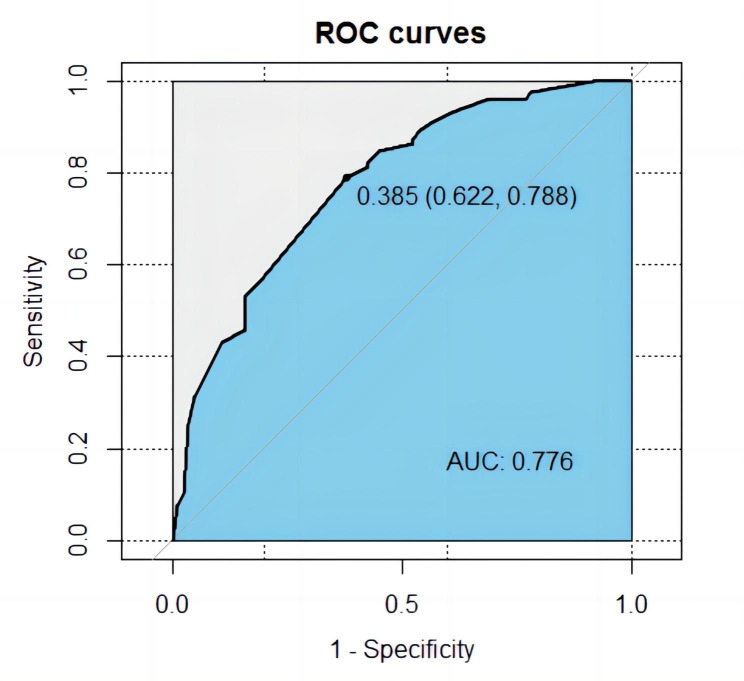
ROC curve for the crescent formation nomogram prediction in the cohort. ROC curve. It showed that the AUC of this model for predicting crescent formation was 0.776. The optimal cut-off value of the ROC curve was 0.385, corresponding to a specificity and sensitivity of 0.622 and 0.788, respectively

The calibration plot of the model demonstrated that high consistence between prediction of crescent formation and actual observation **(**Fig. 4**)**. According to ROC curve, the AUC value was 0.776 (95%CI 0.622–0.788) **(**Fig. 5**)**, indicated that the model has medium discrimination. The C-index of the prediction model was 0.776 (95%CI 0.711–0.840). We further performed internal validation on the nomogram by bootstrapping validation, and calculated revised C-index as 0.755, which indicated that the model had a relatively great predictive discrimination.

### Clinical use of the nomogram

**Fig. 6:**
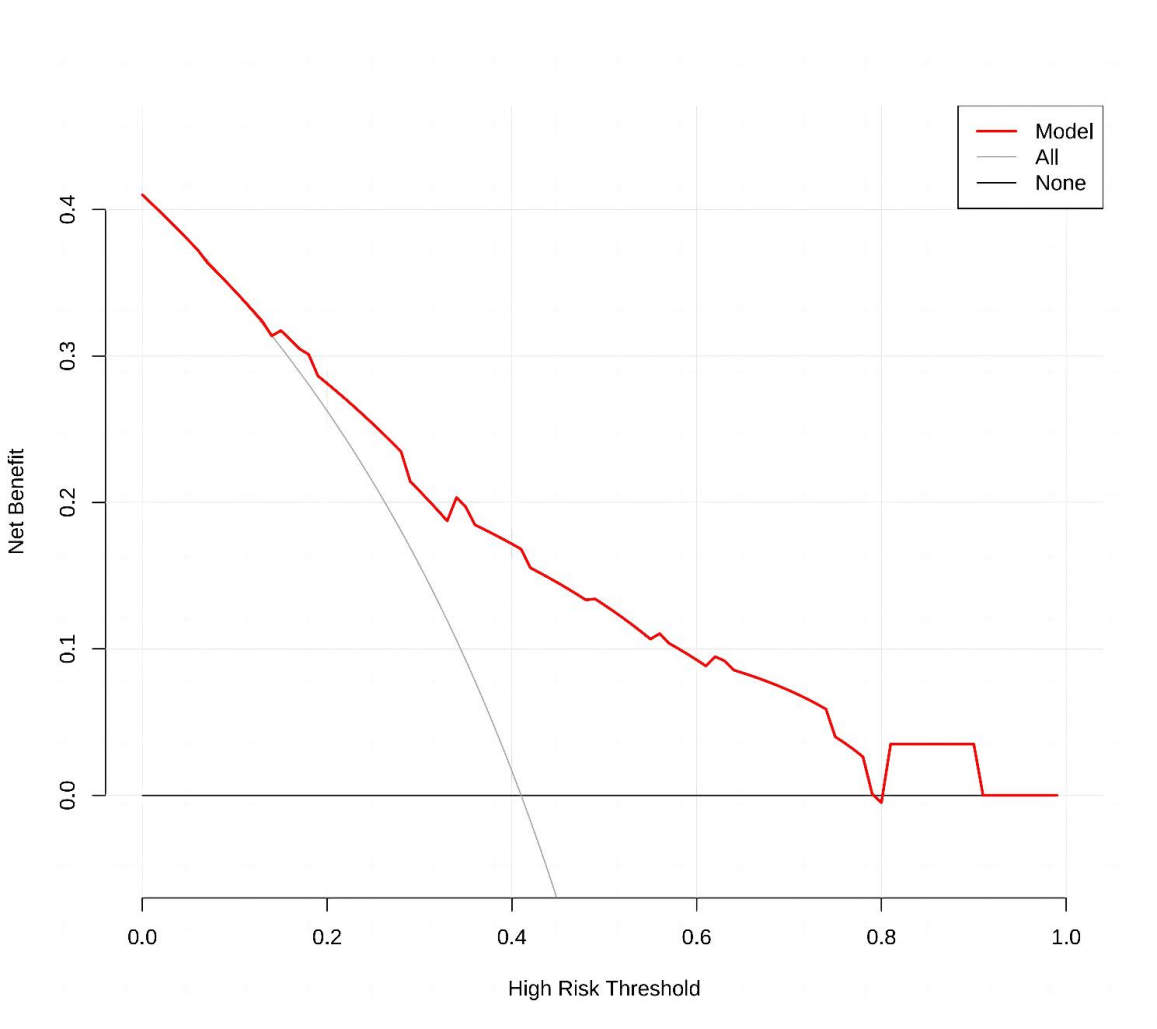
Decision curve analysis (DCA) for the crescent formation nomogram. DCA. The y-axis measures the net benefit. The red solid line represents the crescent formation nomogram. The solid grey line represents the assumption that all patients with lgA nephropathy developed crescent formation. The solid black line represents the assumption that none of the patients with lgA nephropathy developed crescent formation. The decision analysis curve showed that the net benefit rate was > 0 at the high-risk threshold of 1–80%, which was clinically significant

In addition to ROC analysis, DCA has been increasingly used to demonstrate the clinical efficacy in clinical models. The DCA for the crescent formation incidence risk nomogram is shown in Fig. 6. The decision curve showed that using the nomogram model to predict the occurrence of crescent formation had a higher net income at a high-risk threshold between 1 and 80%.

## Discussion

Crescents are lesions commonly identified by renal biopsy in IgAN patients, which indicate active and severe glomerular injury. Patients with crescents were found to have more clinicopathologic risk factors for poor prognosis of IgAN [19, 20]. In this study, we established a new tool for predicting the risk of crescent formation in IgA nephropathy to guide clinical management. The nomogram is an intuitive graphical prediction model that can be used to predict certain clinical outcomes or rates of adverse events [21]. The C-index of internal verification showed that the model had a good discrimination and calibration capabilities [22]. In addition, we analyzed ROC curves to validate this nomogram model and evaluated the clinical application value of the prediction tool through DCA, which suggested the satisfactory performance of our model. The nomogram suggests that urinary protein ≥ 1 g, URBC counts ≥ 30/ul, mALBU ≥ 1500 mg/L, eGFR < 60ml/min/1.73m^2^, and serum IgA/C3 ratio ≥ 2.59 may be used as the independent factors that increase the risk of crescent formation.

In this study, we found that urinary protein ≥ 1 g was an independent predictor of crescent formation in patients with IgAN, which is similar to some previous studies [23, 24]. Zhang et al. [25] retrospectively analyzed 538 IgAN patients with different proportions of crescents and found that the increasing crescent proportion was associated with an increased amount of urine protein excretion. The above results indicated a synergistic effect between proteinuria and crescent formation. An earlier study found that extensive changes in podocytes were observed during the formation of crescents [26]. Podocytes further induced the proliferation of parietal epithelial cells by adhering to the glomerular basement membrane and parietal basement membrane to form cell crescent, resulting in the damage of the glomerular filtration membrane and proteinuria [27, 28].

Microscopic hematuria is one of the common clinical manifestations of IgAN. Previous studies have found that a large amount of microscopic hematuria could indicate active renal inflammation and thus may be used as a surrogate marker of crescent [29, 30]. Our results indicated that URBC counts ≥ 30/ul were an independent risk factor for crescent formation in IgAN patients. Nagai et al. [31] found that the possibility of crescents in the severe hematuria group (URBCs ≥ 30/HPF) was significantly increased (OR = 4.3, 95%CI 1.7–10.9). In addition, in vitro studies confirmed that glomerular vascular injury and GBM breaks cause plasma leakage, which triggers cellular and fibrocellular crescent formation [32].

Our results showed that serum IgA/C3 ratio ≥ 2.59 is remarkably related to crescent formation. Previous studies have shown that the serum IgA/C3 ratio is an auxiliary diagnostic marker of IgAN, which can predict the severity of clinicopathology and prognosis of IgAN patients [33, 34]. Kawasaki et al. [35] reported that crescents and mesangial hypercellularity score in IgAN patients with a high serum IgA/C3 ratio and strong glomerular C3 staining were higher than those in IgAN patients with a low serum IgA/C3 ratio and weak glomerular C3 staining. It has been speculated that there is complement during the formation of IgAN crescents and that serum IgA/C3 ratio reflects the degree of pathological kidney damage. Moreover, Itami et al. [36] found that the average immunofluorescence scores of renal IgA, MASP2, and kappa of IgAN patients with crescent were significantly higher than those without crescent; it has been suggested that complement participates in the formation of crescents after activation through the MBL pathway. However, this value may only apply to IgAN patients from this cohort and requires external validation in other ethnic groups, or in countries with multi-ethnic populations or different biopsy practices.

In this prediction model, eGFR < 60ml/min/1.73m^2^ is recognized as the important risk factor for crescents formation. Shao et al. [37] performed a meta-analysis to evaluate the clinical and prognostic significance of crescent formation and found that IgAN patients with crescents exhibit lower eGFR levels. Sun et al. [38] reported that an increasing fraction of glomeruli with crescents was associated with a reduced eGFR. The reason for this phenomenon might be that the impact of crescents on nephron depends on the feature and proportion of the crescent [39]. The fading of single nephron glomerular filtration rate was initiated by the gradually increasing counter pressure in Bowman’s capsule and may endorse the collapse of the glomerular tuft. Futhermore, once a glomerular crescent involves and obstructs the urinary pole, the entire nephron no longer contributes to total GFR [40].

In addition, Our results demonstrated that IgAN patients have a higher risk of crescent formation when they present with mALBU ≥ 1500 mg/L. Microalbuminuria as an early protein detection index has been widely carried out and applied to the early assessment of renal damage, especially in the early evaluation of diabetic nephropathy and hypertensive kidney damage [41, 42]. A cross-sectional study of Wu et al. [43] found urinary protein components such as urinary ALB, IgG and Trf can reflect severity of pathological lesions in IgAN. Guo et al. [44] further confirmed that crescent is a risk factor to affect prognosis of IgAN patients with microalbuminuria. This strong correlation between them suggests that we should pay attention to IgAN patients with microalbuminuria in our clinical work. Routine screening for microalbuminuria may help to identify those with crescent formation in whom early therapeutic interventions could reduce the risk of developing progressive renal disease.

There are several limitations in the present study. First, the number of cases investigated was small since it was a single-center study and the cohort was not representative of all Chinese patients with IgAN. In the future, we will expand the number of patients in subsequent studies to analyze the prediction model. Although the sample size of this cohort was relatively small, this prediction model still is expected to provide reliable estimates. The reasons are as follows:1) Multivariate regression was carried out only after the independent variables were screened. Since there were many variables and few samples, we used lasso regression to screen the sample size. 2) The OR values and confidence intervals of the results are relatively normal. 3) The goodness of fit results of the model show that the model modeling is successful(Cox-Snell R^2^ = 0.218, R^2^_Nagelkerke_ = 0.293). Second, the analysis of risk factors did not include all potential factors affecting the formation of crescents, such as drug treatment, inflammatory cytokines, and other factors, were not thoroughly evaluated. Third, the nomogram lacks external validation in other IgAN populations from other regions and countries and may require further external validation by a multicenter sample study.

## Conclusions

Based on the five risk factors of urinary protein ≥ 1 g, URBC counts ≥ 30/ul, mALBU ≥ 1500 mg/L, eGFR < 60ml/min/1.73m^2^, and serum IgA/C3 ratio ≥ 2.59, a nomogram model was constructed to predict the risk of crescent formation in IgAN patients. The model had high accuracy, discrimination, and predictive ability, indicating its potential practicability for high-risk patients’ clinical screening and medical intervention.

## Data Availability

The datasets used and/or analysed during the current study are available from the corresponding author on reasonable request.

## List of abbreviations

IgAN: IgA nephropathy
ESRD: End-stage renal disease
MAP: Mean arterial pressure
URBC: Urinary red blood cell
eGFR: Estimated glomerular filtration rate
BP: Blood pressure
LDL: Low-density Lipoprotein
HDL: High-density Lipoprotein
IgA: serum immunoglobulin A
C3: serum complement 3
IgGU: urine immunoglobulin G, β2-MgU:urine β2-microglobulin
mALBU: urine micro-albumin
α1-MgU: urine α1-microglobulin
α2-MgU: urine α2-microglobulin
TrfU: Urinary transferrin
M: Mesangial hypercellularity
E: endocapillary hypercellularity
S: Segmental glomerulosclerosis
T: Tubular atrophy/interstitial fibrosis
C: Crescents
C-index: Harrell’s concordance index
AUC: Area under the curve

## References

[1] Tomino Y. New Insights into the pathogenesis and treatment of patients with immunoglobulin A Nephropathy[J]. Journal of Experimental & Clinical Medicine; 2012.

[2] Xie J, Lv J, Wang W, Li G, Liu Z, Chen H (2018). Kidney failure risk prediction equations in IgA nephropathy: a Multicenter Risk Assessment Study in Chinese Patients.[J]. Am J Kidney Diseases: Official J Natl Kidney Foundation.

[3] Yeter HH, Gonul I, Guz G, Helvaci O, Korucu B, Akcay OF, et al. Combining clinical features and MEST-C score in IgA nephropathy may be a better determinant of kidney survival.[J]. Romanian journal of internal medicine = Revue roumaine de medecine interne; 2020. 10.2478/rjim-2020-0025 32841168

[4] Xu R, Li Z, Cao T, Xu Y, Liao Y, Song H (2021). The Association of the Oxford classification score with longitudinal estimated glomerular filtration rate decline in patients with immunoglobulin A nephropathy: a mixed-method study.[J]. Int J Gen Med.

[5] Shi SF, Wang SX, Jiang L, Lv JC, Liu LJ, Chen YQ (2011). Pathologic predictors of renal outcome and therapeutic efficacy in IgA nephropathy: validation of the oxford classification.[J]. Clin J Am Soc Nephrology: CJASN.

[6] Zhang X, Shi S, Ouyang Y, Yang M, Shi M, Pan X (2018). A validation study of crescents in predicting ESRD in patients with IgA nephropathy.[J]. J Translational Med.

[7] Park S, Baek CH, Park SK, Kang HG, Hyun HS, Park E (2019). Clinical significance of Crescent formation in IgA Nephropathy - a Multicenter Validation Study.[J]. Kidney Blood Press Res.

[8] Neves P, Pinheiro RBB, Dias CB, Yu L, Testagrossa LA, Cavalcante LB, et al. Renal outcomes in brazilian patients with Immunoglobulin A Nephropathy and Cellular Crescentic Lesions[J]. Volume 45. Kidney & Blood Pressure Research; 2020. pp. 1–11. 3. 10.1159/000507251 32299081

[9] Haas M, Verhave JC, Liu ZH, Alpers CE, Barratt J, Becker JU (2017). A Multicenter Study of the Predictive Value of Crescents in IgA Nephropathy.[J]. J Am Soc Nephrology: JASN.

[10] Trimarchi H, Barratt J, Cattran DC, Cook HT, Coppo R, Haas M et al. Oxford classification of IgA nephropathy 2016: an update from the IgA nephropathy classification Working Group.[J]. 2017,91(5):1014. 10.1016/j.kint.2017.02.003 28341274

[11] Lees JS, McQuarrie EP, Mordi N, Geddes CC, Fox JG, Mackinnon B (2017). Risk factors for bleeding complications after nephrologist-performed native renal biopsy[J]. CKJ: Clin Kidney J.

[12] Lin SY, Chang CY, Lin CC, Hsu WH, Liu IW, Lin CD et al. Complications of Outpatient and Inpatient Renal Biopsy: A Systematic Review and Meta-Analysis.[J]. Diagnostics (Basel, Switzerland), 2021,11(4). 10.3390/diagnostics11040651 PMC8066170 33916860

[13] Stevens LA, Claybon MA, Schmid CH, Chen J, Horio M (2011). Evaluation of the chronic kidney disease epidemiology collaboration equation for estimating the glomerular filtration rate in multiple ethnicities.[J]. Kidney Int.

[14] DeMers D, Wachs D, Physiology. Mean Arterial Pressure[J]. 2023. 30855814

[15] Riley RD, Snell KI, Ensor J, Burke DL, Harrell FE, Moons KG (2019). Minimum sample size for developing a multivariable prediction model: PART II - binary and time-to-event outcomes.[J]. Stat Med.

[16] Riley RD, Van Calster B, Collins GS (2021). A note on estimating the Cox-Snell R from a reported C statistic (AUROC) to inform sample size calculations for developing a prediction model with a binary outcome.[J]. Stat Med.

[17] Pate A, Riley RD, Collins GS, van Smeden M, Van Calster B, Ensor J (2023). Minimum sample size for developing a multivariable prediction model using multinomial logistic regression.[J]. Stat Methods Med Res.

[18] Lee MJ, Kim SJ, Oh HJ, Ko KI, Koo HM, Kim CH (2014). Clinical implication of crescentic lesions in immunoglobulin A nephropathy.[J]. Nephrology, dialysis, transplantation: official publication of the european Dialysis and Transplant Association. - Eur Ren Association.

[19] Ma F, Liu L, Dong R, Yang X, Wei L, Li L et al. Renal survival and risk factors in IgA nephropathy with crescents[J]. Int Urol Nephrol, 2020(5). 10.1007/s11255-020-02457-3 32533530

[20] Ruan Y, Hong F, Wu J, Lin M, Wang C, Lian F, et al. Clinicopathological characteristics, risk factors and renal outcome in IgA nephropathy with crescents.[J]. Journal of nephrology; 2022. 10.1007/s40620-022-01273-5 35290652

[21] Zhou H, Zhang Y, Qiu Z, Chen G, Hong S, Chen X (2018). Nomogram to Predict cause-specific mortality in patients with surgically resected Stage I non–small-cell lung Cancer: a competing risk Analysis[J]. Clin Lung Cancer.

[22] Wei L, Champman S, Li X, Li X, Li S, Chen R et al. Beliefs about medicines and non-adherence in patients with stroke, diabetes mellitus and rheumatoid arthritis: a cross-sectional study in China[J]. Bmj Open, 2017,7. 10.1136/bmjopen-2017-017293 PMC5640055 28982826

[23] Le W, Liang S, Hu Y, Deng K, Bao H, Zeng C (2012). Long-term renal survival and related risk factors in patients with IgA nephropathy: results from a cohort of 1155 cases in a chinese adult population.[J]. Nephrology, dialysis, transplantation: official publication of the european Dialysis and Transplant Association. - Eur Ren Association.

[24] Canney M, Barbour SJ, Zheng Y, Coppo R, Zhang H, Liu ZH (2021). Quantifying duration of Proteinuria Remission and Association with Clinical Outcome in IgA Nephropathy.[J]. J Am Soc Nephrology: JASN.

[25] Zhang W, Zhou Q, Hong L, Chen W, Yang S, Yang Q (2017). Clinical outcomes of IgA nephropathy patients with different proportions of crescents[J]. Medicine.

[26] Hir ML, Keller C, Eschmann V, Hähnel B, Hosser H, Kriz W (2001). Podocyte bridges between the tuft and Bowman’s capsule: an early event in experimental crescentic glomerulonephritis[J]. J Am Soc Nephrol.

[27] Bariéty J, Bruneval P, Meyrier A, Mandet C, Hill G, Jacquot C (2005). Podocyte involvement in human immune crescentic glomerulonephritis.[J]. Kidney Int.

[28] Sicking EM, Fuss A, Uhlig S, Jirak P, Dijkman H, Wetzels J (2012). Subtotal ablation of parietal epithelial cells induces crescent formation.[J]. J Am Soc Nephrology: JASN.

[29] Gowrishankar S, Gupta Y, Vankalakunti M, Gowda KK, Kurien AA, Jansi Prema KS (2019). Correlation of Oxford MEST-C Scores with clinical variables for IgA Nephropathy in South India.[J]. Kidney Int Rep.

[30] Bobart SA, Alexander MP, Shawwa K, Vaughan LE, Ghamrawi R, Sethi S (2021). The association of microhematuria with mesangial hypercellularity, endocapillary hypercellularity, crescent score and renal outcomes in immunoglobulin A nephropathy.[J]. Nephrology, dialysis, transplantation: official publication of the european Dialysis and Transplant Association. - Eur Ren Association.

[31] Nagai M, Kobayashi N, Izumi N, Ohbayashi T, Hotta O, Hamano T. Pre-treatment hematuria and crescents predict estimated glomerular filtration rate trajectory after methylprednisolone pulse therapy with tonsillectomy for IgA nephropathy.[J]. Journal of nephrology; 2021. 10.1007/s40620-021-01064-4 34014510

[32] Ryu M, Migliorini A, Miosge N, Gross O, Shankland S, Brinkkoetter PT (2012). Plasma leakage through glomerular basement membrane ruptures triggers the proliferation of parietal epithelial cells and crescent formation in non-inflammatory glomerular injury.[J]. J Pathol.

[33] Mizerska-Wasiak M, Małdyk J, Rybi-Szumińska A, Wasilewska A, Miklaszewska M, Pietrzyk J et al. Relationship between serum IgA/C3 ratio and severity of histological lesions using the Oxford classification in children with IgA nephropathy[J]. Pediatric Nephrology, 2015,30(7). 10.1007/s00467-014-3024-z 25549975

[34] Stefan G, Stancu S, Boitan B, Zugravu A, Petre N, Mircescu G (2020). Is there a role for IgA/C3 ratio in IgA Nephropathy Prognosis? An outcome analysis on an european Population.[J]. Iran J Kidney Dis.

[35] Kawasaki Y, Maeda R, Ohara S, Suyama K, Hosoya M (2018). Serum IgA/C3 and glomerular C3 staining predict severity of IgA nephropathy.[J]. Pediatr Int.

[36] Itami H, Hara S, Samejima K, Tsushima H, Morimoto K, Okamoto K, et al. Complement activation is associated with crescent formation in IgA nephropathy[J]. Archiv Für Pathologische Anatomie Und Physiologie Und Für Klinische Medicin; 2020. 10.1007/s00428-020-02800-0 32300880

[37] Shao X, Li B, Cao L, Liang L, Yang J, Wang Y (2017). Evaluation of crescent formation as a predictive marker in immunoglobulin A nephropathy: a systematic review and meta-analysis[J]. Oncotarget.

[38] Sun Q, Yu D, Chen H, Zhu B, Hu Y, Jiang F et al. Clinicopathological features and prognosis of IgA nephropathy with different proportions of crescentic lesions. Journal of Wenzhou Medical University, 2016,47(3):201–205. 2016.

[39] Yang D, Liu H, Peng Y, Fu Y, Chen A, Xu X (2021). Clinical implication of the circumferential crescents lesions in immunoglobulin A nephropathy: a single-center study of Han Chinese population.[J]. Hum Pathol.

[40] Anguiano L, Kain R, Anders HJ (2020). The glomerular crescent: triggers, evolution, resolution, and implications for therapy.[J]. Curr Opin Nephrol Hypertens.

[41] Mulè G, Castiglia A, Cusumano C, Scaduto E, Geraci G, Altieri D (2017). Subclinical kidney damage in hypertensive patients: a renal window opened on the Cardiovascular System. Focus on Microalbuminuria [J] Advances in Experimental Medicine and Biology.

[42] Thethi TK, Batuman V (2019). Challenging the conventional wisdom on diabetic nephropathy: is microalbuminuria the earliest event?[J]. J Diabetes Complicat.

[43] WU J, Xie Y, Yin Z, Zhang X, Chen X, Nephrology W. Analysis the relationship between urinary protein components and clinical-pathological characteristics in IgA Nephropathy Patients. 2009.

[44] Guo Z, Wang Y, Li H, Li X, Wu Y (2015). Risk factor analysis of prognosis of IgA nephropathy with microalbuminuria. J Chin Physician.

